# *β*-Asarone Increases Chemosensitivity by Inhibiting Tumor Glycolysis in Gastric Cancer

**DOI:** 10.1155/2020/6981520

**Published:** 2020-04-10

**Authors:** Heyun Tao, Xuelian Ding, Jian Wu, Shenlin Liu, Wei Sun, Mengjun Nie, Xiaoting Pan, Xi Zou

**Affiliations:** ^1^The Affiliated Hospital of Nanjing University of Chinese Medicine, Jiangsu Province Hospital of Chinese Medicine, Nanjing 210029, Jiangsu, China; ^2^No. 1 Clinical Medical College, Nanjing University of Chinese Medicine, Nanjing 210023, Jiangsu, China

## Abstract

*β*-asarone is the main active ingredient of the Chinese herb *Rhizoma Acori Tatarinowii,* which exhibits a wide range of biological activities. It was confirmed to be an efficient cytotoxic agent against gastroenteric cancer cells. However, the exact mechanism of *β*-asarone in gastric cancer (GC) remains to be elucidated. The present study showed the inhibitory effect of *β*-asarone on three types of different differentiation stage GC cell lines (MGC803, SGC7901, and MKN74) in a dose-dependent manner. Meanwhile, the synergistic sensitivity of *β*-asarone and cisplatin was confirmed by using the median-effect principle. Flow cytometry assay revealed that under both normoxia and CoCl_2_-induced hypoxia conditions, *β*-asarone can induce apoptosis of GC cells, which can block GC cells in the cell cycle G2/M phase, showing obvious subdiploid peak. Moreover, the activity of lactic dehydrogenase (LDH), an enzyme that plays an important role in the final step of tumor glycolysis, was significantly decreased in GC cells following treatment with *β*-asarone. Mechanistically, *β*-asarone can reduce pyruvate dehydrogenase kinase (PDK) 1, phospho(p)-PDK1, PDK4, hypoxia-inducible factor 1-*α* (HIF1*α*), c-myc, STAT5, and p-STAT5 expression, which revealed how *β*-asarone affects tumor glycolysis. In conclusion, the present study provided evidence in support of the hypothesis that the increase of chemotherapy sensitization by *β*-asarone is associated with the inhibition of tumor glycolysis.

## 1. Introduction

Approximately 70% of the energy required by the human body is produced by glucose metabolism, which provides support for various biochemical reactions. Glycolysis and mitochondrial oxidative phosphorylation (OXPHOS) are the two major glucose metabolic pathways responsible for generating ATP. Normal cells generate energy mainly via the OXPHOS pathway under physiological oxygen conditions as it is a more efficient metabolic process than glycolysis. Tumor cells are characterized by metabolic reprogramming, among which differential hydrolysis of glucose is the most prominent and earliest confirmed metabolic abnormality [1]. The German biochemist Otto Warburg demonstrated for the first time that even under aerobic conditions, cancer cells mainly produce a large amount of lactic acid through glycolysis instead of mitochondrial oxidative glycolysis. This phenomenon is called tumor aerobic glycolysis, also known as the Warburg effect [2–4]. This metabolic shift towards increased glycolytic functioning sustains tumor cell proliferation and aggressiveness [5].

Although glycolysis has a lower capacity in producing ATP, it can lead to chemotherapy resistance of tumor cells through a number of ways [6, 7]. In addition, solid tumors form an anoxic environment through glycolysis, in which there are a large number of hypoxic cells. Studies show that these hypoxic cells are more resistant to radiation than aerobic cells, while less sensitive to chemotherapeutic drugs [8]. Therefore, in the application of conventional radiotherapy and chemotherapy dose therapy, hypoxic cells cannot be effectively killed, which becomes one of the important factors of refractory tumor resistance [9]. Therefore, this metabolic transformation of tumor cells may help to develop novel anticancer drugs and formulate new treatment strategies to overcome the resistance of tumor cells to current treatment options [10, 11].

Pyruvate dehydrogenase kinases (PDKs) are pivotal enzymes in the glucose metabolism pathway. Glucose is metabolized to pyruvate, which is transformed into acetyl-CoA by the pyruvate dehydrogenase complex (PDC) in order for mitochondrial respiration in normal cells to occur with sufficient oxygen. In cancer cells, forced expression of PDKs could inactivate PDC by phosphorylation of PDC [12], thereby shunting the pyruvate away from the OXPHOS by retarding its conversion to acetyl-CoA. Emerging evidence demonstrated that the expression levels of PDKs were elevated in various types of cancers, such as breast cancer [13], gastric cancer [14], glioblastoma [15], hepatocellular carcinoma [16], and melanoma [17], and were closely associated with poor tumor prognosis.

The main base material of Rhizoma Acori Graminei of Araceae is a volatile oil, while *β*-asarone is the main effective component of volatile oil [18]. It is reported that *β*-asarone has a variety of biological activities, such as antiepileptic, antithrombotic, insecticidal, and bactericidal effects and myocardial protection [19]. Jian Wu et al. [20, 21] found that *β*-asarone showed a positive proliferation inhibitory effect on various digestive tract tumor cells. Although the antitumor effects of *β*-asarone have been verified, its specific mechanism of action remains to be elucidated. In the present study, the differences in the chemosensitivity of GC cells before and after *β*-asarone treatment were compared, and the underlying mechanisms were investigated. The results showed that *β*-asarone could increase cisplatin chemotherapy sensitization and reverse tumor glucose metabolic disturbance in cancer cells. Further investigation verified that the mechanism is related to inhibiting the expression of PDKs in GC cells.

## 2. Materials and Methods

### 2.1. Cell Culture

Three types of cell lines, low-differentiated human GC cell line MGC803, moderately differentiated human GC cell line SGC7901, and highly differentiated human GC cell line MKN74, were obtained from the Type Culture Collection, Chinese Academy of Sciences (Shanghai, China) and were routinely cultured in RPMI-1640 medium containing 10% bovine serum, penicillin (100 U/ml), and streptomycin (100 *μ*g/ml) at 37°C with 5% CO_2_.

### 2.2. Drugs and Reagents

*β*-asarone, dimethyl sulfoxide (DMSO), and cisplatin were purchased from Sigma-Aldrich (St. Louis, MO, USA). RPMI-1640 medium, 0.25% trypsin, penicillin-streptomycin, phosphate buffer saline (PBS), and anti-HIF-1*α*, PDK4 antibodies were purchased from Gibco BRL (Gaithersburg, MD, USA) and 3-(4,5-dimethylthiazol-2-yl)-2, 5-diphenyltetrazolium bromide (MTT) from Biosharp Biological Technology (Hefei, China). Bovine serum was purchased from Sijiqing Biological Engineering Materials Co., Ltd (Hangzhou, China) and Annexin V/propidium iodide (PI) apoptosis detection kit from KeyGen Biotech Co., Ltd. (Nanjing, China). DNA content quantitation assay kit was purchased from Youningwei Biotechnology Co., Ltd. (Shanghai, China). Primescript reverse transcription reagent kit with gDNA Eraser and SDS sample buffer was purchased from TaKaRa (Dalian, China) and TRIzol reagent and Power SYBR-Green PCR Master Mix from Life Technologies (Grand Island, NY). Primary anti-PDK1 (#3062), p-PDK1 (#3061), STAT5 (#9310), p-STAT5 (#9351), *β*-actin (#4967) antibodies and secondary antibodies antirabbit IgG HRP (#7074) and antimouse IgG HRP (#7076) were products of Cell Signaling Technology Inc. (Beverly, MA, USA). All of the reagents were analytically pure.

### 2.3. MTT Assay

The experimental groups were divided into four groups: *β*-asarone group, cisplatin group, *β*-asarone + cisplatin group, and control group. Cells in the logarithm phase were seeded in 96-well plates at the density of 3.5 × 10^3^ (MGC803), 4 × 10^3^ (SGC7901), 5 × 10^3^ (MKN74)/well, respectively. After the cells adhered for 24 h, the medium in the plate was discarded, then the cells were incubated with different concentrations of different drugs mixed with RPMI-1640 medium at 37°C with 5% CO_2_ for 24 h, followed by adding 20 *μ*l MTT (5 g/l) per well and incubating for 4 h. Subsequently, the supernatant was removed and 150 *μ*l dimethylsulfoxide (DMSO) was added. Cells were then solubilized in DMSO. Absorbance under 490 nm was detected to calculate the inhibition rate, and the same experiment was repeated 3 times. Inhibition rate (% of control) = (1 − absorbance of test sample/absorbance of control) × 100%.

### 2.4. Combined Drug Evaluation

After the treatment of five different concentration gradients of different groups, the median-effect principle was used to analyze the interaction between *β*-asarone and cisplatin. Coadministration of *β*-asarone and cisplatin was evaluated using the following formulas: fa = 1 − (OD experiment/OD control), fa/fu = (D/Dm) m, where fa means cell inhibition rate, fu means cell survival rate, D means the concentration of drugs, m means slope, and Dm is median concentration. Then according to the above formula, CI was calculated to evaluate the interaction between *β*-asarone and cisplatin, CI = D1/Dx1 + D2/Dx2. D1 and D2 mean the respective concentrations may be needed when having x effect by coadministration of *β*-asarone and cisplatin while Dx1 and Dx2 mean the concentration of *β*-asarone or cisplatin when having x effect. When CI < 1, combined drugs have a synergistic action, CI > 1 means one drug antagonizes another.

### 2.5. LDH Assay

Human GC cells in a logarithm growth phase were inoculated into a 6-well plate at the density of 3.5 × 10^3^ (MGC803), 4 × 10^3^ (SGC7901), and 5 × 10^3^ (MKN74)/ml, respectively, and incubated for 24 h. Then the medium was removed and the mixed solution with *β*-asarone at a concentration of 0, 7.5, 15, 30, 60, or 120 *μ*g·ml^−1^ was added to the 6-well plate. Twenty-four hours later, supernatant was collected. Standards were made according to the manufacture' s instructions. 50 *μ*l standard and 50 *μ*l samples were both added to plates, followed by adding antibody with biotin and incubating for 1 h, at 37°C. The ELISA plate was filled with detergent, which was removed after 30 s shaking, and patting it dry with bibulous paper, repeated 3 times. Afterward, cells were incubated for 30 min, at 37°C with 80 *μ*l streptavidin per well. Incubation was carried out with 50 *μ*l·A and 50 *μ*l·B for 10 min, at 37°C, in a dark place. The ELISA plate was subjected to 50 *μ*l stoping solution per well. Optical density (OD) was detected under 450 nm to calculate the density of LDH.

According to the preliminary experiment results, Cocl_2_ (200 *μ*M, 24 h) was used to induce hypoxia conditions [22] while H_2_O_2_ (100 *μ*M, 1 h) was used to induce peroxide condition. Under these conditions, the viability of the cells will not be greatly affected. Therefore, the above experiment was repeated which were grouped as follows: Cocl_2_ (200 *μ*M, 24 h) group, Cocl_2_ (200 *μ*M, 24 h) + *β*-asarone (60 *μ*g·ml^−1^, 24 h) group, *β*-asarone (60 *μ*g·ml^−1^, 24 h) group, H_2_O_2_ (100 *μ*M, 1 h) group, H_2_O_2_ (100 *μ*M, 1 h) + *β*-asarone (60 *μ*g·ml^−1^, 24 h) group, and control group.

### 2.6. Cell Apoptosis Assay

Three types of GC cells were plated into a 6-well plate at a density of 2 × 10^5^/well. After adhering for 24 h, the cells were treated with or without *β*-asarone (60 *μ*g·ml^−1^), together with CoCl_2_ (200 *μ*M) or H_2_O_2_ (100 *μ*M) for 24 h, about 5 × 10^5^ cells were harvested and washed twice with cold PBS, resuspended in 500 binding buffer (10 mM Hepes/NaOH, pH 7.4, 140 mM NaCl, 2.5 mM CaCl_2_). About 5 *μ*l Annexin V and 5 *μ*l PI were then added to the solution, and the cells were gently vortexed and incubated for 15 min at room temperature in the absence of light. Apoptosis of the cells was detected by flow cytometry within 1 h. The results were analyzed and quantified by a flow cytometer (BD Biosciences, San Jose, CA, USA).

### 2.7. Cell Cycle Assay

Three types of GC cells were plated into a 6-well plate at a density of 2 × 10^5^/well. After adhering for 24 h, the cells were treated with *β*-asarone in environments with different oxygen concentrations for 24 h, washed twice by cold PBS, and resuspended in 1 ml PBS, to which 4 ml 95% ethyl alcohol was added. Cells were washed with cold PBS twice again after being fixed for 12 h, resuspended in 0.4 ml prepared propidium iodide subsequently, and then incubated for 30 min at 37°C in the dark. Afterward, the cells were kept in 4°C in the absence of light for flow cytometry to analyze cell cycle distributions.

### 2.8. Western Blot Analysis

After the treatment of *β*-asarone in environments with different oxygen concentrations, gastric cancer cells were lysed with RIPA lysis buffer containing protease cocktail (Roche, Mannheim, Germany) on ice for 30 min. The cell lysate was harvested and centrifugated at 12,000 ×*g* for 5 min, and the supernatant was collected. The protein concentrations were determined by the Bradford assay (Bio-Rad, Philadelphia, PA, USA). Twenty micrograms per sample was subjected to SDS-PAGE and then transferred onto polyvinylidene difluoride membranes (Millipore, Billerica, MA, USA). After blocking the nonspecific binding site on the membrane with 5% nonfat milk solution, the membranes were incubated with specific primary antibodies at a dilution of 1 : 1,000 at 4°C overnight. After washing three times with TBS-Tween-20, the membranes were incubated with HRP-conjugated secondary antibody at a dilution of 1 : 2,000 at room temperature for 1 h, and then the bands on the membrane were visualized using an enhanced chemiluminescence reagent (Thermo Fisher Scientific, Inc., Waltham, MA, USA).

### 2.9. RNA Purification and Real Time PCR (RT-PCR)

Having been incubated with *β*-asarone in environments with different oxygen concentrations for 24 h, the human GC cell lines were assessed by RT-PCR to determine the effect of *β*-asarone on the mRNA expression of tumor glycolysis associated genes. Total RNA was isolated by TRIzol reagent. Subsequently, 1 *μ*g of the extracted total RNA was reverse-transcribed into first-strand complementary DNA (cDNA) using the High-Capacity cDNA Reverse Transcription kit according to the manufacturer's instructions. They were then performed on 7500 Fast RT-PCR System, using DNA-binding dye SYBR-Green. The ΔΔCt method was used for qPCR. Primer Premier software (version 3.0; Premier biosoft, California, USA) was used for designing primers of different exons by retrieving NCBI GenBank database and used *β*-actin as an internal control gene. The sequences of the primers used to specifically amplify the genes of interest are shown in Table 1.

### 2.10. Statistical Analysis

SPSS software (version 13.0; IBM SPSS, Armonk, NY, USA) was used for statistical analysis. Student's *t*-test or one-way ANOVA was adopted to evaluate statistical significance. *P* < 0.05 indicated a statistically significant difference.

## 3. Results

### 3.1. *β*-Asarone and Cisplatin Can Suppress GC Cells Proliferation In *Vitro* When Used Independently or in Combination

**Figure 1:**
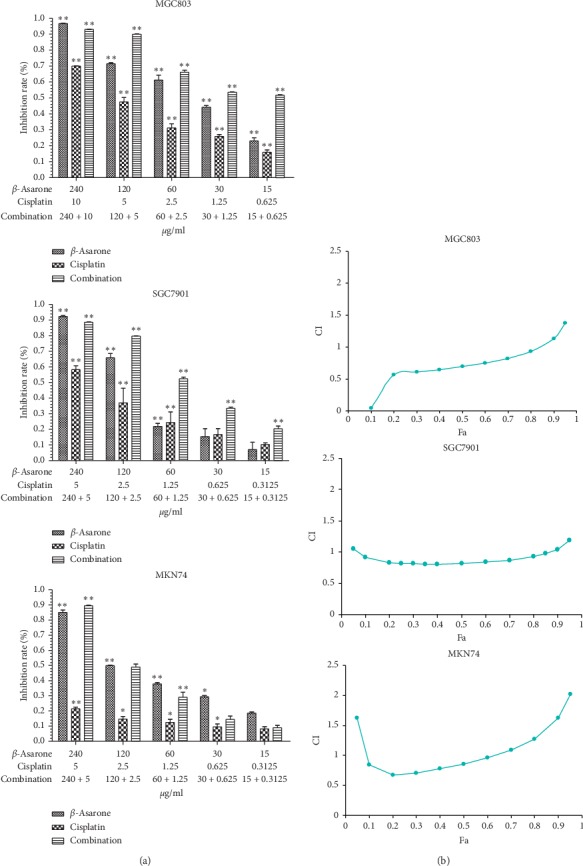
*β*-Asarone inhibits gastric cancer cell proliferation and increases chemosensitivity. (a) Single usage of *β*-asarone or cisplatin and combining usage mediated cell proliferation inhibition with gradient concentrations for 24 h. IC50 values of each drug were following measured. (b) Median-effect principle was used to determine the synergistic effect of combining usage. fa-CI curve graph of MGC803 (upper), SGC7901 (medium), and MKN74 (lower) showed a different section of fa when CI < 1.The results of three independent experiments are expressed as mean ± SD, ^*∗*^*P* < 0.05; ^*∗∗*^*P* < 0.01 (Student's *t*-test) indicate a significant difference vs. the control.

Firstly, the inhibitory effects of *β*-asarone and cisplatin on three different GC cell lines were tested. The results showed that *β*-asarone showed no cytotoxicity at low concentrations (15 *μ*g·ml^−1^) to cell lines with the exception of MGC803 cells. Cell viability was significantly inhibited with increasing concentrations (30–240 *μ*g·ml^−1^) on all three cell lines (Figure 1(a)). The IC50 of *β*-asarone in MGC803, SGC7901, and MKN74 cells was 39.92, 84.6, and 96.22 *μ*g·ml^−1^, respectively (data not shown). Subsequently, the antiproliferative activity of GC cells in the combination group was detected. Based on the results, compared to the IC50 values of cisplatin (4.77, 4.24, and 100.43 *μ*g·ml^−1^ in MGC803, SGC7901, and MKN74 cells, respectively), in the combination usage group, the dose of cisplatin in the *β*-asarone combination group required for half of the cells to die was only 0.79 *μ*g·ml^−1^ in MGC803, 0.99 *μ*g·ml^−1^ in SGC7901, and 1.65 *μ*g·ml^−1^ in MKN74 cells, which is lower compared with the dose when cisplatin is used alone. These data demonstrated that *β*-asarone and cisplatin, when used alone or in combination, could inhibit GC cell proliferation *in vitro*. *β*-asarone also attenuated cisplatin-induced toxicity in GC cells.

### 3.2. The Combination of *β*-Asarone and Cisplatin Has a Synergistic Effect on GC Cells

To investigate objective synergistic or antagonistic effects of the combination of *β*-asarone and cisplatin *in vitro*, the median-effect principle was applied to calculate drug combination indexes according to MTT assay results. As is shown in Figure 1(b), a synergistic effect was observed in MGC803, SGC7901, and MKN74 cells when the numeric value of fa was less than 0.85, 0.07–0.86, and 0.1–0.6, respectively. These results demonstrated that *β*-asarone resulted in chemotherapeutic sensitization in gastric cancer therapy. However, further studies will need to confirm both the safety and dose of *β*-asarone that will provide the most beneficial antitumor effect *in vivo.*

### 3.3. *β*-Asarone Can Suppress GC Cells by Attenuating the Tumor Glycolysis Pathway

**Figure 2:**
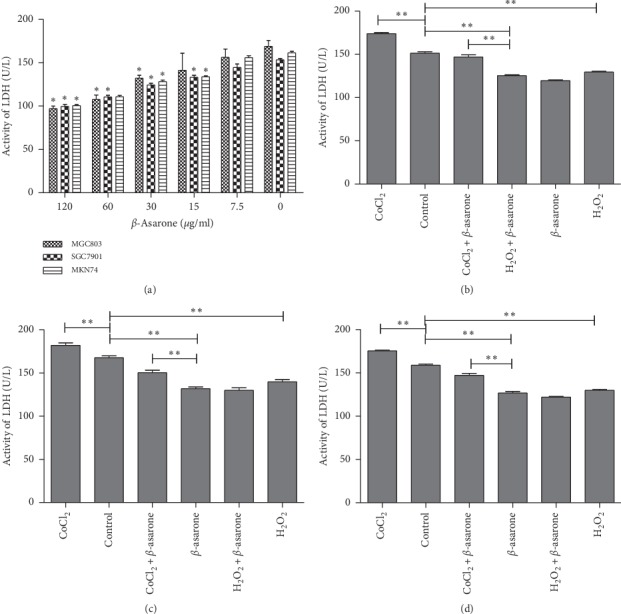
*β*-Asarone inhibits LDH secretion in GC cells in a different environment. (a) After treatment with various concentrations of *β*-asarone, the LDH secretion of GC cells was analyzed as described. (b) MGC803, (c) SGC7901, (d) MKN74. The concentration of LDH was detected after GC cells were treated with *β*-asarone (60 *μ*g·ml^−1^) in different oxygen environments. The results of three independent experiments expressed as mean ± SD are shown. ^*∗*^*P* < 0.05 (Student's *t*-test) indicates a significant difference vs. the control.

First, to confirm the effect of *β*-asarone on tumor glycolysis, LDH activity in GC cells was determined. As is shown in Figure 2(a), following treatment with *β*-asarone at concentrations higher than 30 *μ*g·ml^−1^, the LDH activity in GC cells significantly decreased in a dose-dependent manner (*P* < 0.01). In MGC803 cells, no significant change in LDH activity was observed at *β*-asarone treatment lower than 15 *μ*g·ml^−1^. Meanwhile, in SGC7901 and MKN74 cells, LDH production was still markedly inhibited following treatment with 7.5 *μ*g·ml^−1^*β*-asarone (*P*=0.011 and *P*=0.013, respectively).

Next, hypoxic and peroxidic environments were simulated in GC cells by using CoCl_2_ (200 *μ*M, 24 h) and H_2_O_2_ (100 *μ*M, 1 h), respectively. Compared with the control group, LDH activity was elevated in the CoCl_2_ group and was inhibited in the H_2_O_2_ group, which illustrated that CoCl_2_-induced hypoxia conditions and H_2_O_2_-induced peroxide conditions participated in the alteration of the tumor glycolysis pathway. Therefore, the CoCl_2_ group was used as the negative control group and the H_2_O_2_ group was used as the positive control group in the following experiment. From these data, we can observe that *β*-asarone served a similar inhibition effect on tumor glycolysis with H_2_O_2_. Compared to the use *β*-asarone or H_2_O_2_ alone, no significant difference was observed in the combination group. However, following *β*-asarone treatment, a significant inhibitory effect of LDH was observed in a hypoxic environment (Figures 2(b)–2(d)). This indicated that hypoxia could enhance the Warburg effect, whereas *β*-asarone can reverse this facilitative effect. In addition, no additional or synergistic effect was observed between *β*-asarone and H_2_O_2_. Therefore, we hypothesized that *β*-asarone can suppress GC cells by attenuating the tumor glycolysis pathway, which is more likely in solid tumors marked by hypoxia.

Based on the correlation analysis results of GC cells with different degrees of differentiation, it was concluded that there is no correlation between the variation in LDH content and the degree of cell differentiation (Table 2).

### 3.4. *β*-Asarone Significantly Induces the Apoptosis of Gastric Cancer Cells in a Hypoxic Environment

**Figure 3:**
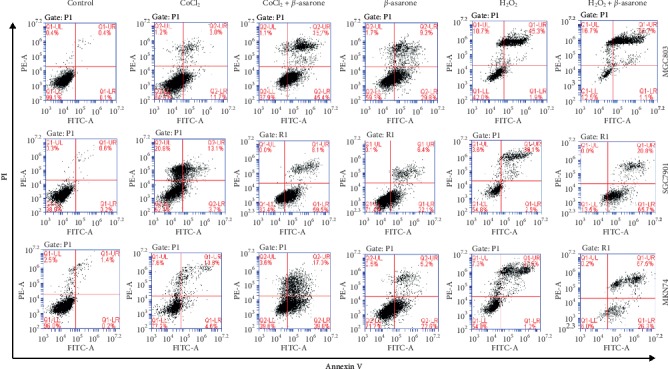
*β*-Asarone induces the apoptosis of gastric cancer cells in the hypoxic environment. Flow cytometric analysis of human gastric cancer cell apoptosis following treatment with *β*-asarone for 24 h. The dual parameter dot plots combining Annexin V-FITC and PI fluorescence showed the viable cell population in the lower left quadrant (Annexin V-PI-), the early-stage apoptotic cells in the lower right quadrant (Annexin V + PI-), and the late-stage apoptotic or dead cells in the upper right quadrant (Annexin V + PI+).

The distinction of apoptosis rates of GC cells under different oxygen environments was determined using an Annexin V/PI double staining assay. The apoptosis rate of the CoCl_2_ group was 14.7% (MGC803), 16.8% (SGC7901), and 15.4% (MKN74). Meanwhile, in the H_2_O_2_ group, the apoptosis rate was 47.2% (MGC803), 41.6% (SGC7901), and 38.7% (MKN74). Following *β*-asarone treatment in normoxic conditions (60 *μ*g·ml^−1^, 24 h), the apoptosis rate was 39% (MGC803), 28.5% (SGC7901), and 27.8% (MKN74). Compared with the positive control and negative control groups, the *β*-asarone effect tends to be in the positive control group. In hypoxic conditions, following treatment with *β*-asarone, the apoptosis rate increased to 61.1% (MGC803), 67.6% (SGC7901), and 56.8% (MKN74). Therefore, it was speculated that *β*-asarone exerted better effects in solid tumors under the hypoxic environment (Figure 3).

**Figure 4:**
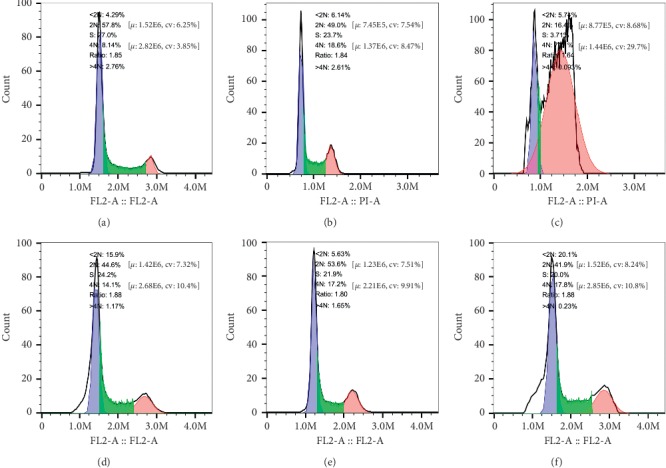
*β*-Asarone blocks GC cells in the cell cycle G2/M phase as well as showing obvious subdiploid peak. Cell cycle distributions of MGC803 were analyzed by flow cytometry after being treated with *β*-asarone under different oxygen conditions; the part before the blue area is subdiploid peak, read area is G2/M phase. 2N is G0/G1 phase, S is S phase, 4N is G2/M phase. (a) Control, (b) CoCl_2_, (c) CoCl_2_ + *β*-asarone, (d) *β*-asarone, (e) H_2_O_2_, and (f) H_2_O_2_ + *β*-asarone.

**Figure 5:**
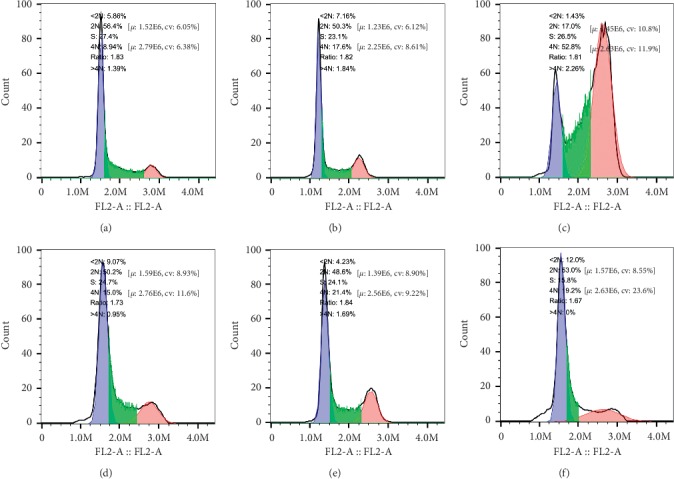
*β*-Asarone blocks GC cells in the cell cycle G2/M phase as well as showing obvious subdiploid peak. Cell cycle distributions of SGC7901 were analyzed by flow cytometry after treated with *β*-asarone under different oxygen conditions; the part before the blue area is subdiploid peak, read area is G2/M phase. 2N is G0/G1 phase, S is S phase, 4N is G2/M phase. (a) Control, (b) CoCl_2_, (c) CoCl_2_ + *β*-asarone, (d) *β*-asarone, (e) H_2_O_2_, and (f) H_2_O_2_ + *β*-asarone.

**Figure 6:**
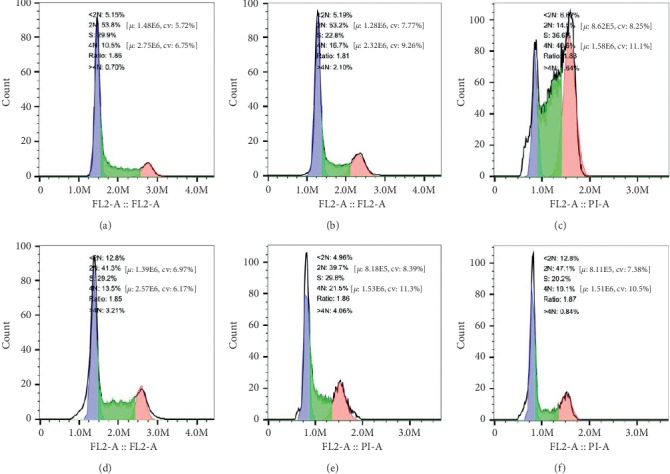
*β*-Asarone blocks GC cells in the cell cycle G2/M phase as well as showing obvious subdiploid peak. Cell cycle distributions of MKN74 were analyzed by flow cytometry after treated with *β*-asarone under different oxygen conditions; the part before the blue area is subdiploid peak, read area is G2/M phase. 2N is G0/G1 phase, S is S phase, 4N is G2/M phase. (a) Control, (b) CoCl_2_, (c) CoCl_2_ + *β*-asarone, (d) *β*-asarone, (e) H_2_O_2_, and (f) H_2_O_2_ + *β*-asarone.

Three different varieties of GC cells were treated with different drugs for 24 h, and the effect on the cell cycle was detected by flow cytometric analysis. As shown in Figures 4–6, compared with the control group, there was no obvious addition of cells in the G0/G1 phase observed in the experimental group, while cells in the G2/M phase were significantly increased. Concurrently, the *β*-asarone-treated group showed obvious subdiploid peaks. These results further suggested that *β*-asarone induced the apoptosis of GC cells.

### 3.5. Changes in the mRNA and Protein Expression of Tumor Glycolysis-Related Genes

**Figure 7:**
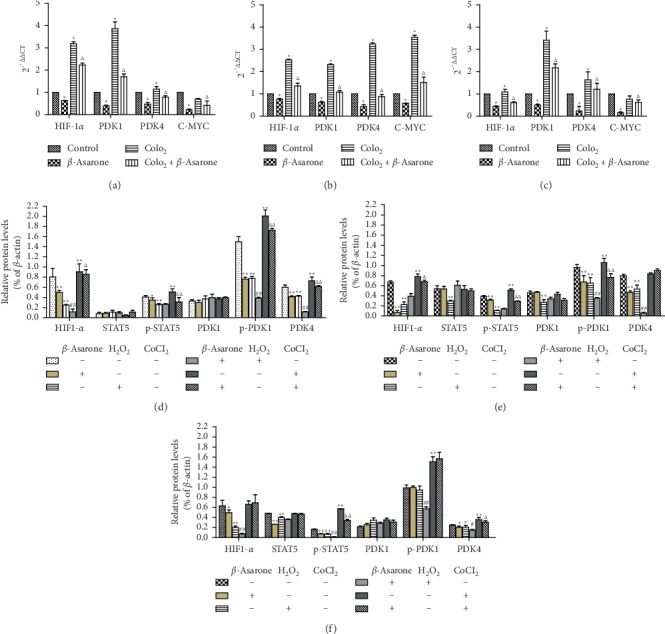
After (a) MGC803, (b) SGC7901, and (c) MKN74 cells were treated with *β*-asarone (60 *μ*g·ml^−1^) and CoCl_2_ (200 *μ*M) single or combined for 24 h the mRNA levels of tumor glycolysis related genes (HIF-1*α*, PDK1, PDK4, and c-myc) were determined by RT-PCR. *β*-Actin was taken as a loading control. (^*∗*^*P* < 0.05, compared with the control group; ^Δ^*P* < 0.05, compared with CoCl_2_ group). (d) MGC803, (e) SGC7901, (f) MKN74. Western blotting analysis of tumor glycolysis related proteins after GC cells were treated with *β*-asarone (60 *μ*g·ml^−1^), CoCl_2_ (200 *μ*M), and H_2_O_2_ (100 *μ*M) single or combined for 24 h (^*∗*^*P* < 0.05, ^*∗∗*^*P* < 0.01, compared with control group; ^#^*P* < 0.05, ^##^*P* < 0.01, compared with H_2_O_2_ group; ^Δ^*P* < 0.05, ^ΔΔ^*P* < 0.01, compared with CoCl_2_ group).

To investigate the mechanism of *β*-asarone action on the tumor glycolysis of GC cells, RT-PCR and western blotting were performed to analyze changes in the mRNA and protein levels, respectively. The results of RT-PCR showed that the expression of HIF-1*α* increased in the hypoxic environment. In addition, after 24 h of incubation of GC cells with *β*-asarone (60 *μ*g·ml^−1^), the mRNA expression levels of HIF-1*α*, PDK1, PDK4 and c-myc were reduced (Figures 7(a)–7(c)).

**Figure 8:**
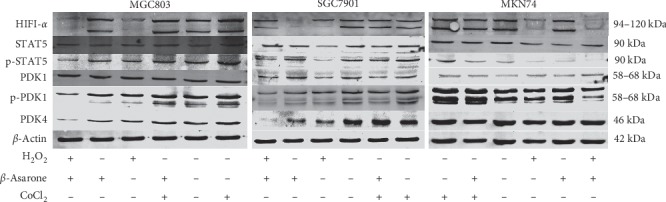
Western blotting was used to determine the expression changes of tumor glycolysis related proteins (HIF-1*α*, STAT5, p-STAT5, PDK1, p-PDK1, and PDK4) after GC cells were treated with different drugs as described. *β*-Actin was used as an internal control.

We next determined the protein expression of HIF-1*α*, STAT5, p-STAT5, PDK1, p-PDK1, and PDK4 in GC cells following treatment with different drugs. Western blotting revealed that the expression level of these tumor glycolysis-related proteins was not completely consistent in all cell lines as well as their changes after being treated by the drugs. In this experiment, we used the routinely cultured cells without any treatment as the control group. The CoCl_2_ group was used as the negative control group, and the H_2_O_2_ group was used as the positive control group. For HIF-1*α* in MGC803 and SGC7901 cells, as well as PDK4 in MGC803 cells and MKN74 cells, the expression was increased in the negative control group while reduced in the *β*-asarone group and positive control group, compared with the control group. Moreover, compared with the negative control group, protein expression was reduced in the CoCl_2_ + *β*-asarone group. However, for HIF-1*α* in MKN74 cells and PDK4 in SGC7901 cells, no matter in the negative control group or following treated with *β*-asarone, no significant changes were observed in the protein level. The STAT5 and phosphorylated STAT5 expression were next detected. Results showed p-STAT5 expression in all three cell lines was reduced in the *β*-asarone group and positive control group and was increased in the negative control group, compared with the control group. Similarly, its expression was reduced in the CoCl_2_ + *β*-asarone group, compared with the negative control group. At the same time, STAT5 expression did not change significantly after being treated with *β*-asarone except in MKN74 cells. For PDK1 and p-PDK1, the same phenomenon as STAT5 and p-STAT5 was also observed in MGC803 and SGC7901 cells. However, the protein levels of p-PDK1 in MKN74 cells did not significantly change after the treatment of *β*-asarone. Compared to the H_2_O_2_ group, the H_2_O_2_ + *β*-asarone group did not show a significant difference in most of the protein expression. Hence, no additional or synergistic effect was observed between *β*-asarone and H_2_O_2_ (Figures 8, 7(d)–7(f)). These data further illustrated how *β*-asarone acts on tumor glycolysis of GC cells. Moreover, the potential molecular mechanism may not be consistent in cell lines with different degrees of differentiation. Combined with RT-PCR results, we can conclude that the hypoxia condition simulated by CoCl_2_ in MKN74 cells does not regulate HIF-1*α* protein translation as well as PDK4 in SGC7901 cells. CoCl_2_-upregulated expression of HIF-1*α* in MKN74 cells and PDK4 in SGC7901 cells most likely occurs only at the transcription. Data about PDK1 and STAT5 indicate that PDK1 may participate in glycolysis through phosphorylation as well as STAT5 in all three cells. While in MKN74 cells, *β*-asarone may not function through PDK1 posttranslational modification of phosphorylation.

## 4. Discussion

The latest tumor epidemiology research suggested that GC remains a refractory tumor worldwide and is the fifth most frequently diagnosed cancer and the third leading cause of cancer deaths [23]. Although therapeutic strategies, including targeted therapy, are constantly improving, chemotherapy still plays a pivotal role in GC therapy. Platinum drugs play an important role in the treatment of GC [24]. However, drug resistance is an existing long-term problem. Studies [25] have shown that most patients obtain resistance within one year of using platinum drugs. Therefore, ways to improve the sensitivity of chemotherapy have become the focus of the current study.

In the present study, using an *in vitro* cell proliferation assay, we demonstrated that *β*-asarone could inhibit GC cells (MGC803, SGC7901, and MKN74) with different degrees of differentiation. A significant synergistic effect (CI < 1) between *β*-asarone and cisplatin in a certain concentration range was observed by the median-effect principle. On this basis, we further explored the molecular mechanism behind the chemosensitivity of *β*-asarone.

Tumor glycolysis is now recognized as a hallmark of cancer [26], and the role of tumor glycolysis in chemotherapy resistance has been illustrated in various cancers, such as glioma, prostatic adenocarcinoma, and cervical carcinoma [27]. Glycolysis resistance to radiation therapy was also observed [28]. However, the strong dependence of cancer cells on glycolysis could be a defect. Consequently, targeting tumor glycolysis is gaining traction in reversing chemotherapy resistance in cancer cells, including novel drugs in the different preclinical and clinical study phases for intracellular targets [29, 30].

LDH catalyzes the conversion of pyruvic acid to lactic acid in the tumor glycolysis pathway. The concentration and action time of CoCl_2_ and H_2_O_2_ that showed minimal impact on cell viability was selected for subsequent experiments. The results showed that LDH activity was upregulated in oxygen-deficient conditions simulated by CoCl_2_, and significantly downregulated after combination with *β*-asarone treatment. In peroxide conditions simulated by H_2_O_2_, LDH activity was downregulated. Following addition with *β*-asarone, LDH activity did not show any significant difference. These findings demonstrated that lack of oxygen can enhance tumor glycolysis, and *β*-asarone can significantly reverse this potentiation. Suppression of lactic dehydrogenase expression could inhibit the production of lactic acid or making it back to mitochondria to improve mito-OxPhos, and TCA may cause the inhibition of aerobic glycolysis [31]. As shown by the anticancer effect of dichloroacetate (DCA) [32], this reversion, from reduction metabolism to oxidation metabolism, brings out a promising result. Additionally, when cells retain OxPhos capacity, pyruvic acid can be oxidized via the mitochondria, leading to an increase in ROS production [33]. The long-term accumulation of ROS promotes cell stress, which results in cell death. This is consistent with our findings in the following experiments. Many factors can impact the process of cell apoptosis. In the present study, there is also an apoptosis rate at about 15% in the CoCl2 group as CoCl2 is a chemical anoxic inducer which can induce cell apoptosis by participating in the MAPK signaling pathway [21]. Meanwhile, the apoptosis rates of the H_2_O_2_ group increased, which also confirmed the relationship between the Warburg effect and cell viability. Compared with the *β*-asarone and CoCl_2_ groups, the apoptosis rate obviously increased and can block cells in the G2/M phase when *β*-asarone is added under oxygen-deficient conditions. However, it is worth noting that not all aerobic glycolysis processes in tumor cells can be reversed, and the reversion may promote tumor cell mobility and metastasis [34].

Gene and protein detection demonstrated that the inhibition of glycolysis by *β*-asarone was mainly attributed to the suppression of the PDK expression. The regulation of PDK overexpression in tumor cells is complex. MYC was identified to cooperate with hypoxia-inducible factor 1-*α* (HIF1-*α*) to induce the expression of PDK1, which inactivates PDC and diminishes mitochondrial OXPHOS [35]. Following induction of PDK1, p-PDK1, which act as multiple AGC protein kinase families involved in the glycolytic pathway and regulating physiological processes such as cell proliferation and antiapoptosis, can be formed by phosphorylation of serine in 241 sites. The experiments demonstrated that in MGC803 and SGC7901 cells, the expression of HIF1-*α*, c-myc and p-PDK1 can be inhibited with *β*-asarone treatment, while the protein levels of PDK1 did not significantly change, indicating that PDK1 may participate in glycolysis through phosphorylation and was regulated by HIF1-*α* and c-myc. While in MKN74 cells, *β*-asarone may not function through PDK1 posttranslational modification of phosphorylation. The effect of signal transduction and STAT were emphasized in recent research [36]. Therefore, we investigated the levels of STAT5 protein, which is an upstream target gene of c-myc. The results showed that phosphorylated STAT5 was inhibited with *β*-asarone treatment. Hence, glycolysis can be inhibited by the regulation of c-myc. Of course, the in-depth molecular mechanisms need to be further explored in this base.

In conclusion, based on the Warburg effect, we proposed and tested a new Warburg destructive treatment, which can be combined with traditional cytotoxic therapies. To the best of our knowledge, this is the first study to illustrate the effect of *β*-asarone in chemosensitivity and the potential mechanism, which may be related to the inhibition of tumor aerobic glycolysis. This finding may potentially contribute to GC treatment.

## Figures and Tables

**Table 1:** Sequences of the primers used in the RT-PCR amplification.

Gene primer	Sequence (5′-3′)	
ACTB	F	GGCCAACCGCGAGAAGAT
	R	CGTCACCGGAGTCCATCA
PDK4	F	GTACAGTTGACCCAGTCACCAA
	R	CCACATCACAGTTAGGATCAATG
HIF-1*α*	F	GGCAGCAACGACACAGAAACTGA
	R	TTGGCGTTTCAGCGGTGGGT
PDK1	F	CACCAGGACAGCCAATACAAGT
	R	ATCCTCATTACCCAGCGTGAC
c-myc	F	GAAACGACGAGAACAGTTGAAA
	R	CAAGGTTGTGAGGTTGCATTT

**Table 2:** The correlation analysis between the differentiated degree of GC cells and LDH secretion.

			Cell	LDH
Rho of Spearman	Cell	Correlation coefficient	1.000	−0.033
		Sig. (two-side)	—	0.810
		*N*	54	54
	LDH	Correlation coefficient	−0.033	1.000
		Sig. (two-side)	0.810	—
		*N*	54	54

## Data Availability

The data in this article are authentic, valid, and available.

## References

[1] Jain M., Nilsson R., Sharma S. (2012). Metabolite profiling identifies a key role for glycine in rapid cancer cell proliferation. *Science*.

[2] Warburg O., Wind F., Negelein E. (1927). The metabolism of tumors in the body. *The Journal of General Physiology*.

[3] Pedersen P. L. (2007). Warburg, me and Hexokinase 2: multiple discoveries of key molecular events underlying one of cancers’ most common phenotypes, the “Warburg effect”, i.e., elevated glycolysis in the presence of oxygen. *Journal of Bioenergetics and Biomembranes*.

[4] Koppenol W. H., Bounds P. L., Dang C. V. (2011). Otto Warburg’s contributions to current concepts of cancer metabolism. *Nature Reviews Cancer*.

[5] Icard P., Shulman S., Farhat D., Steyaert J.-M., Alifano M., Lincet H. (2018). How the Warburg effect supports aggressiveness and drug resistance of cancer cells?. *Drug Resistance Updates*.

[6] Holohan C., Van Schaeybroeck S., Longley D. B., Johnston P. G. (2013). Cancer drug resistance: an evolving paradigm. *Nature Reviews Cancer*.

[7] Raz S., Sheban D., Gonen N., Stark M., Berman B., Assaraf Y. G. (2014). Severe hypoxia induces complete antifolate resistance in carcinoma cells due to cell cycle arrest. *Cell Death & Disease*.

[8] Clarke R. H., Moosa S., Anzivino M. (2014). Sustained radiosensitization of hypoxic glioma cells after oxygen pretreatment in an animal model of glioblastoma and in vitro models of tumor hypoxia. *PLoS One*.

[9] Levine A. J., Puzio-Kuter A. M. (2010). The control of the metabolic switch in cancers by oncogenes and tumor suppressor genes. *Science*.

[10] Cairns R. A., Harris I. S., Mak T. W. (2011). Regulation of cancer cell metabolism. *Nature Reviews Cancer*.

[11] Granchi C., Fancelli D., Minutolo F. (2014). An update on therapeutic opportunities offered by cancer glycolytic metabolism. *Bioorganic & Medicinal Chemistry Letters*.

[12] McFate T., Mohyeldin A., Lu H. (2008). Pyruvate dehydrogenase complex activity controls metabolic and malignant phenotype in cancer cells. *Journal of Biological Chemistry*.

[13] Sutendra G., Dromparis P., Kinnaird A. (2012). Mitochondrial activation by inhibition of PDKII suppresses HIF1a signaling and angiogenesis in cancer. *Oncogene*.

[14] Hur H., Xuan Y., Kim Y. B. (2013). Expression of pyruvate dehydrogenase kinase-1 in gastric cancer as a potential therapeutic target. *International Journal of Oncology*.

[15] Jha M. K., Suk K. (2013). Pyruvate dehydrogenase kinase as a potential therapeutic target for malignant gliomas. *Brain Tumor Research and Treatment*.

[16] Shen Y.-C., Ou D.-L., Hsu C. (2013). Activating oxidative phosphorylation by a pyruvate dehydrogenase kinase inhibitor overcomes sorafenib resistance of hepatocellular carcinoma. *British Journal of Cancer*.

[17] Abildgaard C., Dahl C., Basse A. L., Ma T., Guldberg P. (2014). Bioenergetic modulation with dichloroacetate reduces the growth of melanoma cells and potentiates their response to BRAFV600E inhibition. *Journal of Translational Medicine*.

[18] Liu L., Fang Y.-Q., Xue Z.-F., He Y.-P., Fang R.-M., Li L. (2012). Beta-asarone attenuates ischemia-reperfusion-induced autophagy in rat brains via modulating JNK, p-JNK, Bcl-2 and Beclin 1. *European Journal of Pharmacology*.

[19] Geng Y., Li C., Liu J. (2010). Beta-asarone improves cognitive function by suppressing neuronal apoptosis in the beta-amyloid hippocampus injection rats. *Biological & Pharmaceutical Bulletin*.

[20] Zou X., Liu S.-L., Zhou J.-Y., Wu J., Ling B.-F., Wang R.-P. (2012). Beta-asarone induces LoVo colon cancer cell apoptosis by up-regulation of caspases through a mitochondrial pathway in vitro and in vivo. *Asian Pacific Journal of Cancer Prevention*.

[21] Wu J., Zhang X.-X., Sun Q.-M. (2015). *β*-asarone inhibits gastric cancer cell proliferation. *Oncology Reports*.

[22] Guan F., Schaffer L., Handa K., Hakomori S.-i. (2010). Functional role of gangliotetraosylceramide in epithelial-to-mesenchymal transition process induced by hypoxia and by TGF-*β*. *The FASEB Journal*.

[23] Bray F., Ferlay J., Soerjomataram I., Siegel R. L., Torre L. A., Jemal A. (2018). Global cancer statistics 2018: GLOBOCAN estimates of incidence and mortality worldwide for 36 cancers in 185 countries. *CA: A Cancer Journal for Clinicians*.

[24] Reed E., Chabner B. A., Chabner B. A., Longo D. L. (2011). Platinum analogues. *Cancer Chemotherapy and Biotherapy: Principles and Practice*.

[25] Perego P., Robert J. (2016). Oxaliplatin in the era of personalized medicine: from mechanistic studies to clinical effificacy. *Cancer Chemotherapy and Pharmacology*.

[26] Hanahan D., Weinberg R. A. (2011). Hallmarks of cancer: the next generation. *Cell*.

[27] Wartenberg M., Richter M., Datchev A. (2010). Glycolytic pyruvate regulates P-glycoprotein expression in multicellular tumor spheroids via modulation of the intracellular redox state. *Journal of Cellular Biochemistry*.

[28] Bhatt A. N., Chauhan A., Khanna S. (2015). Transient elevation of glycolysis confers radio-resistance by facilitating DNA repair in cells. *BMC Cancer*.

[29] Hay N. (2016). Reprogramming glucose metabolism in cancer: can it be exploited for cancer therapy?. *Nature Reviews Cancer*.

[30] Gill K. S., Fernandes P., O’Donovan T. R. (2016). Glycolysis inhibition as a cancer treatment and its role in an anti-tumour immune response. *Biochimica et Biophysica Acta (BBA)-Reviews on Cancer*.

[31] Marchiq I., Albrengues J., Granja S., Gaggioli C., Pouyssegur J., Simon M. P. (2015). Knock out of the BASIGIN/CD147 chaperone of lactate/H+ symporters disproves its pro-tumour action via extracellular matrix metalloproteases (MMPs) induction. *Oncotarget*.

[32] Papandreou I., Goliasova T., Denko N. C. (2011). Anticancer drugs that target metabolism: is dichloroacetate the new paradigm?. *International Journal of Cancer*.

[33] Denko N. C. (2008). Hypoxia, HIF1 and glucose metabolism in the solid tumour. *Nature Reviews Cancer*.

[34] Andrzejewski S., Klimcakova E., Johnson R. M. (2017). PGC-1alpha promotes Breast cancer metastasis and confers bioenergetic flflexibility against metabolic drugs. *Cell Metabolism*.

[35] Dang C. V. (2008). The interplay between MYC and HIF in the Warburg effect. *Oncogenes Meet Metabolism*.

[36] Poli V., Camporeale A. (2015). STAT3-mediated metabolic reprograming in cellular transformation and implications for drug resistance. *Frontiers in Oncology*.

